# Child and family experiences with inborn errors of metabolism: a qualitative interview study with representatives of patient groups

**DOI:** 10.1007/s10545-015-9881-1

**Published:** 2015-07-25

**Authors:** Sara D. Khangura, Kylie Tingley, Pranesh Chakraborty, Doug Coyle, Jonathan B. Kronick, Anne-Marie Laberge, Julian Little, Fiona A Miller, John J. Mitchell, Chitra Prasad, Shabnaz Siddiq, Komudi Siriwardena, Rebecca Sparkes, Kathy N. Speechley, Sylvia Stockler, Yannis Trakadis, Brenda J. Wilson, Kumanan Wilson, Beth K. Potter, on behalf of the Canadian Inherited Metabolic Diseases Research Network (CIMDRN)

**Affiliations:** Faculty of Medicine, School of Public Health and Preventive Medicine, University of Ottawa, 451 Smyth Road, Ottawa, ON K1H 8M5 Canada; Newborn Screening Ontario, Ottawa, ON Canada; Division of Metabolics and Newborn Screening, Department of Pediatrics, Children’s Hospital of Eastern Ontario, Ottawa, ON Canada; Faculty of Medicine, Department of Pediatrics, University of Ottawa, Ottawa, ON Canada; Hospital for Sick Children, Toronto, ON Canada; University of Toronto, Toronto, ON Canada; CHU Sainte-Justine, Montréal, QC Canada; Institute of Health Policy, Management and Evaluation, University of Toronto, Toronto, ON Canada; Montréal Children’s Hospital, Montréal, QC Canada; Western University, London, ON Canada; University of Alberta Hospital, Edmonton, AB Canada; Alberta Children’s Hospital, Calgary, AB Canada; University of British Columbia, Vancouver, BC Canada; Ottawa Hospital Research Institute, Ottawa, ON Canada

## Abstract

**Background:**

Patient-centered health care for children with inborn errors of metabolism (IEM) and their families is important and requires an understanding of patient experiences, needs, and priorities. IEM-specific patient groups have emerged as important voices within these rare disease communities and are uniquely positioned to contribute to this understanding. We conducted qualitative interviews with IEM patient group representatives to increase understanding of patient and family experiences, needs, and priorities and inform patient-centered research and care.

**Methods:**

We developed a sampling frame of patient groups representing IEM disease communities from Canada, the United States, and United Kingdom. With consent, we interviewed participants to explore their views on experiences, needs, and outcomes that are most important to children with IEM and their families. We analyzed the data using a qualitative descriptive approach to identify key themes and sub-themes.

**Results:**

We interviewed 18 organizational representatives between February 28 and September 17, 2014, representing 16 IEMs and/or disease categories. Twelve participants voluntarily self-identified as parents and/or were themselves patients. Three key themes emerged from the coded data: managing the uncertainty associated with raising and caring for a child with a rare disease; challenges associated with the affected child’s life transitions, and; the collective struggle for improved outcomes and interventions that rare disease communities navigate.

**Conclusion:**

Health care providers can support children with IEM and their families by acknowledging and reducing uncertainty, supporting families through children’s life transitions, and contributing to rare disease communities’ progress toward improved interventions, experiences, and outcomes.

## Background

Advances in diagnostic and therapeutic interventions for children with inborn errors of metabolism (IEM) are preserving and extending the lives of affected children. However, the impacts of these interventions on the experiences and daily lives of children with IEM and their families are not well understood (Potter et al 2013). While several studies have undertaken quantitative investigation into psychosocial, quality of life, and related outcomes (Cederbaum et al 2001; Fabre et al 2013; Weber et al 2012), fewer have described affected children’s and families’ experiences using a qualitative approach (Packman et al 2010; Vegni et al 2010). Nonetheless, the importance of understanding the patient and family experience as part of providing patient-centered care is gaining recognition (Liberati 2011). This may be of particular importance for rare diseases like IEM, where there is considerable clinical heterogeneity, emphasizing the relevance of personalized care that is tailored to individual needs (Andersson et al 2005).

Patient groups have emerged as leading voices and advocates for patients with rare diseases like IEM and their families (Hall 2013). Often initiated by patients and families themselves, these groups have been instrumental toward defining and furthering the needs and priorities of rare disease patient communities (Dunkle et al 2010). The perspectives of these groups thus promise to lend value to understanding the collective experiences, priorities, and concerns of patients and their families.

The Canadian Inherited Metabolic Diseases Research Network (CIMDRN) is a national network of researchers and collaborators investigating interventions and outcomes for children with IEM and their families (Potter et al 2013). As part of CIMDRN’s research into patient and family experiences, we conducted a qualitative interview study to gain insight from patient groups that support children with IEM and their families. Specifically, we sought perspectives on some of the most important experiences, needs, and priorities of families living with and managing pediatric IEM. In addition to informing patient-centered health care and support services for IEM, we aimed to enrich and advance current understanding of children’s and families’ experiences in order to support patient- and family-oriented outcomes research in the field of IEM.

## Method

### Design, sampling, recruitment, and participants

We sought to conduct qualitative, semi-structured interviews with a maximum of 20–30 participants, or until saturation of concepts and themes (Walker 2012) was reached. We developed a sampling frame of eligible patient groups using suggestions from study investigators, the Canadian Directory of Genetic Support Groups (Canadian Association of Genetic Counsellors n.d.) and targeted internet searches. While eligibility was initially limited to Canadian groups, it was later expanded to include any English-speaking rare disease patient group with a public Internet presence and a focus on one or more IEMs. This expanded eligibility allowed for a larger sampling frame from which to characterize experiences with IEM—an objective that transcends geographic borders. Individual participant eligibility was restricted to those employed by, or working on a volunteer basis for, an eligible group, and who held a spokesperson and/or representative role. The particular eligible individual from the patient group was generally selected by the respondent and/or the patient group.

Recruitment invitations were distributed by email or web contact forms in batches (between 5 and 21 at one time). Eligible participants were invited to participate via e-mail or electronic web forms available on the patient group internet sites. Non-responders received one follow-up invitation. Respondents were asked to provide signed, informed consent to participate in the study and were provided with the proposed interview questions in advance. Telephone interviews were then scheduled, conducted, and audio-recorded. The study protocol was approved by the Ottawa Health Science Network Research Ethics Board (OHSN-REB).

### Data collection

Interviews were conducted by two members of CIMDRN’s research staff (SDK, KT) using a semi-structured interview guide which was informed by a broad scoping review of relevant patient- and family-oriented outcomes (Khangura et al 2015). The guide addressed such topics as quality of life, physical, mental and social health, and health care experiences. The guide was reviewed by study investigators with expertise in the clinical management of IEM and/or qualitative research.

An experienced medical transcriptionist transcribed each audio-recorded interview which was later verified by a researcher (SS) who simultaneously listened to the audio-recording while reading the corresponding transcript. In one instance, the recording device failed; however, this participant had provided unsolicited answers to the questions in the interview guide via e-mail prior to the interview. Thus, we included data from this participant’s e-mail answers.

### Analysis

Given the overarching objectives of this study, the primary analytical approach was qualitative descriptive (Sandelowski 2010). This method is considered appropriate when describing the ‘what’ of phenomena by minimizing interpretation (Neergaard et al 2009; Sandelowski 2000).

Three researchers formed the core analytical team (SDK, BKP, KT) and inductively identified initially broad conceptual categories (sub-themes) using the first four interview transcripts. Specific concepts were subsequently identified, assigned codes, applied by one researcher (SDK) using NVivo software (version 10), and reviewed for credibility and trustworthiness (Shenton 2004) by a second researcher (KT, SS). As the study progressed, and interviews were transcribed and coded in parallel, key themes emerged from the conceptual categories, which were further explored in later interviews. Regular team meetings were held to review findings as they emerged and discuss the extent to which unique conceptual categories or themes were yet being identified, or whether saturation had been reached (Walker 2012), informing decisions concerning further recruitment.

## Results

### Participants

Forty-six recruitment invitations were distributed and 18 group representatives provided signed, informed consent, participating in interviews between February 28, 2014, and September 17, 2014, inclusive. Non-responders provided no information about reasons for non-participation or -response. Interviews ranged in duration from approximately 30 to 60 minutes.

Of the 18 respondents, five were male and most held various executive-level positions within their organizations, i.e., seven were President or Vice President; eight held Directorship positions; and the remaining three were a Board Member, Family Services Manager, and group Founder.

Diseases represented by participating patient groups were as follows:

**Box 1: Diseases represented by 18 participating IEM patient groups (n if >1)**

**Table:** 

alpha-1 antitrypsin deficiency
galactosemia
leukodystrophies
Pompe disease
Fabry disease
glucose transporter type 1 (Glut1) deficiency syndrome
mitochondrial disorders
porphyrias
fatty oxidation disorders
glycoprotein storage diseases
mucopolysaccharidosis (MPS) (2)
rare diseases (answers specific to IEM were sought)
gaucher disease
inborn errors of metabolism (general)
phenylketonuria (PKU) (2)
Wilson disease

Twelve of the 18 participants provided estimates of the number of children and families associated with their group (the remaining six participants could not provide an estimate), which ranged from two to 5000, with a median of 100 children and their families per group. This generally reflected the size of the group, but also the degree to which the disease(s) were identified earlier versus later in life.

While the interviews sought information about affected children and families associated with the group, 12 of the participants self-identified as parents of affected children and/or were themselves patients. Often, these participants volunteered personal insights about living with an IEM. We did not exclude these, nor distinguish between personally lived experiences and perceptions about the experiences of patients and families, as participants themselves did not draw this distinction.

Prior to conduct of the last several interviews, saturation of themes was suspected during a regular team meeting at which the findings from the analyses were assessed. This was confirmed at a follow-up meeting after the 18th interview when the findings from the analysis of the last several interviews were assessed. Recruitment was subsequently ended on September 17, 2014.

#### Child and family experiences with IEM: perspectives of patient groups

Despite heterogeneity within and across diseases in the patient group representatives’ perceptions about the experiences and priorities of patients and their families, we identified three key themes bearing relevance to the full set of 18 interviews: coping with uncertainty and the unknown; the challenges accompanying an affected child’s major life transitions and; the collective struggle for improved outcomes and interventions that rare disease communities navigate. Sub-themes (based on the conceptual categories in the coded data) were also identified and are described as they appear within the key themes.

### Coping with uncertainty and the unknown

This theme emerged generally, as participants described the daily lives and experiences of children and families (Box 2, [1a–b]). Often, the unknown was associated with challenges surrounding the diagnosis—generally a series of intense experiences for families (Box 2, [2a]). Some participants highlighted lengthy delays and/or misdiagnoses as acute exacerbations of distress (Box 2, [2b]), or contributors to a catastrophic outcome (Box 2, [2c]).

Following diagnosis, uncertainty about the child’s prognosis and future was a common experience. For families whose children had diseases with neurocognitive manifestations, for example, challenges dealing with uncertainty regarding how the child would be affected in the long-term were described (Box 2, [3a]). In addition, for those whose children had diseases that were progressive and terminal, uncertainty about the child’s mortality was highlighted (Box 2, [3b]).

Often, uncertainty was made manifest through interactions with the health care system and health care providers. While this was commonly described with respect to primary and emergency care, even among specialist physicians, some participants described perceived uncertainty (Box 2, [4a])—reflecting the many remaining ‘unknowns’ about rare diseases like IEM (Box 2, [4b]). Nonetheless, participants also expressed recognition that health care providers cannot be expected to have an in-depth understanding of all rare diseases (Box 2, [4c]).

Some participants described strategies for managing uncertainty and the unknown, including becoming self-informed (Box 2 [5a]); gathering with other affected families for support and information (Box 2, [5b]) and; educating others about the disease (Box 2, [5c]).

**Box 2: Coping with uncertainty and the unknown—example quotes**

**Table:** 

**Sub-theme 1: Dealing with the unknown (in general)**
**[1a]** Participant (06): *“I think for me* [the most important impact on daily life is] *the unknown. Because like… you don’t know what the future’s going to hold. That’s hard.”*
**[1b]** Participant (12): *“One of the things that people find so difficult to deal with is the uncertainty and the unknowing, sort of thing…”*
**Sub-theme 2: Uncertainty concerning diagnosis**
**[2a]** Participant (03)*: “So, you know there’s the challenges once you get diagnosed, but there’s all those challenges before you were diagnosed, and you know, often people go through a couple of years and several specialists until they get the diagnosis.”*
**[2b]** Participant (17): “*… it’s my feeling that* [the disease] *is a very under-diagnosed rare disease just because of the misdiagnosis for 5 years for my son… .. And there’s so many stories that are even worse than that.”*
**[2c]** Participant (05): *“… quite often infants were actually diagnosed after they had passed away… I can’t imagine being a parent who goes through that.”*
**Sub-theme 3: Unknowns about the child’s prognosis and future**
**[3a]** Participant (06): *“I think just overall just the not knowing what to expect and you’re hoping, you know, like I’m hoping my son gets through high school. And I’m hoping he gets a diploma, and if we can get him there I’ll be thrilled, you know. I’m not looking at college, I’m not looking at university or anything for him, but I’m hoping that maybe he can be responsible enough that he can do something. Maybe in the trades, maybe with his hands, and maybe we can set him up in his own business. So, but that’s kind of a hard reality…”*
**[3b]** Participant (16): “*A lot of people want to know like, ‘When is, you know, when is my child gonna die?’… Some doctors will say, ‘Well you know, a kid with* [the disease] *is gonna die, you know, between the ages of five and 10.’ So then like, every year you know, the birthday is not something that is a happy celebration for these families…* [they] *want to know ‘When is this gonna happen?’ And just the unknowns about that.”*
**Sub-theme 4: Uncertainty among health care providers**
**[4a]** Participant (10): *“Well the primary care system has no idea… And then even in the smaller clinics throughout the country, you know, they may only have one patient with* [the disease]*. And so even though they’re the specialists, they don’t know everything.”*
**[4b]** Participant (01): *“I think there’s a lot to be learned yet… there’s very little research between symptoms—in general, between symptoms and between* [biochemical] *levels.”*
**[4c]** Participant (02): *“I get it; there are 7000 rare diseases, and nobody can be an expert on all of them.”*
**Sub-theme 5: Strategies for coping with the unknown**
**[5a]** Participant (16): *“In most cases the families… very quickly become the expert… I think just trying to understand the disease, becoming the expert and being able to advocate and share and raise awareness about their disease locally is probably the hardest thing…”*
**[5b]** Participant (01): *“I mean at the best, the best we can do at our end is to try to match families together so that they can share information and you know talk to somebody who has the disorder and you know understand what they do and how they cope.”*
**[5c]** Participant (17): *“I go in to my family doctor and whenever he’s got a student in with him we sit down for 15 min and I educate him on* [the disease]*… But this is part of raising awareness, ‘I’ll teach you about a rare disease so that maybe 1 day in your life, if you come across these symptoms you’ll remember’, right?”*

#### Child’s life transitions

Experiences and challenges in the daily management of an affected child’s health and well-being featured prominently in the interviews—particularly those surrounding the child’s life transitions. One of the first of these transitions is entrusting the child’s care to others who may not fully appreciate the gravity of the disease, often at the age when the child begins daycare (Box 3, [1a]). Sometimes, these challenges result in a change to the family dynamic, as a parent may have to give up his or her career to become a full-time caregiver (Box 3, [1b]).

Extending from this was the concept of introducing the affected preschool child to the peer group. Depending on the child and the disease’s manifestations, these were described variably; one self-identified parent of a child with a disease causing immunocompromise described organizing a low-immunity playgroup at her local community center to support her child’s socialization, and the emotional response from one of the mothers in attendance (Box 3, [1c]).

As children transition to school, concerns around the transfer of care, disease management, and social development continue (Box 3, [2a-b]). Likewise, depending on the manifestations of the disease, variable social and emotional challenges were described (Box 3, [2c]).

During the adolescent transition, social challenges were often described as being more acute; one self-identified parent’s adolescent son with a progressive disease described the limitations imposed by the condition and the challenges this introduces for sustaining long-term, meaningful friendships (Box 3, [3a]). On the other hand, adolescents with IEM for which self-management must be actively adopted into adulthood face different social challenges (Box 3, [3b]).

Finally, as adolescents ‘age out’ of pediatric health care, they must navigate health systems that are often perceived as being unprepared for managing those with IEMs who live as long as many now do (Box 3, [3c]).

**Box 3: Child’s life transitions—example quotes**

**Table:** 

**Sub-theme 1: Early childcare and social development**
**[1a]** Participant (08): *“… when you’re also dealing with like daycare providers—and they’ve got a lot of kids they’re dealing with—and how do you make sure that your child is getting what he or she needs at the daycare?”*
**[1b]** Participant (15): *“I know it’s something my family personally has faced; the quandary of needing to work but not being able to because of the severe needs, you know, the high needs and the maintenance needed at home with the condition.”*
**[1c]** Participant (07): *“And this mum… came in crying… I said ‘You know what, like, if this is causing you too much stress…that’s not my intention’… she just kind of burst out crying and hugged me and she said ‘Oh my god, no. I’m so happy,’ she said, ‘my daughter is 4-and-a-half* [years old] *and this is the first time she’s ever been with children’.”*
**Sub-theme 2: Transition to school**
**[2a]** Participant (07): *“You know you’ve just got the daycare all on board* [with managing the disease]*, and you’re done daycare, and now you’re going into the school system. It’s just over, and over, and over, and over again.”*
**[2b]** Participant (15): *“…often* [the symptoms] *our children experience are difficult for others to detect. So that’s always a concern for parents, you know, in the school setting or any kind of setting outside… especially when someone else is filling that caregiver role you know either at school or the parents are at work and things like that.”*
**[2c]** Participant (06): *“… he’ll* [the participant’s affected son] *come home and he’ll say ‘Mummy, I just walked away from them* [other children] *because, you know what? They’re not nice and I don’t need them as friends’, you know… because his speech isn’t where it should be it makes him very hard to understand and that causes a lot of social, you know… no, not social issues; but he couldn’t express himself in the beginning…”*
**Sub-theme 3: Transition through adolescence**
**[3a]** Participant (12): *“One of* [the participant’s son’s] *big challenges—and I think it is for a lot of the kids—is socializing…* [he] *had a really close friend, but unfortunately he moved away. And kids are really good with him, but no one who’ll come over and play. I don’t blame them. I know back when they were 9 or 10 they wanted to play baseball;* [he’s] *not playing baseball. And they’re at the age where they talk really quick and they do things really quick and* [he’s] *in a wheelchair now and, you know. So, like I said they’re always incredibly friendly but there’s nobody who would come over and watch a movie with him.”*
**[3b]** Participant (09): *“I think the only adherence issues we see, as a rule, tends to be at the moment of transition, and the young people being fed up with being told what to do and saying that they’re going to make their own decisions and not adhere to the diet or to the medication because it’s their choice.”*
**[3c]** Participant (13): *“… you know a lot of the children… are now, as it were, the oldest children who’ve ever lived with these conditions… So, there’s no experience of how you provide services and support for people in this position; essentially you’re making it up as you go along…”*

#### Struggle for improved outcomes and interventions

A third, overarching theme was the collective struggle that families living with IEM and their respective disease communities navigate in pursuit of improved interventions and outcomes. While not always stated explicitly, this finding emerged when considering the interviews in juxtaposition with one another. For instance, the concept of access to health care resources was described variably by different group representatives, e.g., while some participants perceived their disease communities as having limited access and/or resources (Box 4, [1a]), others described having celebrated important progress in research and treatment availability (Box 4, [1b]).

Advances in communication—most notably internet and social media—were often credited as an important driver of progress in the struggle for information and interconnectedness. For example, information on the internet was sometimes described as an important resource for the undiagnosed (Box 4, [2a]), or as means for empowering parents with information about their child’s diagnosis (Box 4, [2b]). Most often, participants credited internet and social media with providing improved opportunities to connect with other affected families—effectively reducing geographic distance as a communication barrier and isolating factor (Box 4, [2c]). Though, while the internet has brought progress, it was also acknowledged as a potential source of misinformation (Box 4, [2d]).

Notably, information gathering using the internet was linked to communication with health care providers (Box 4, [3a])—an important part of achieving better outcomes. Some participants described situations where parents necessarily become ‘expert patients’ (by proxy) (Box 4, [3b]), while others offered suggestions for improvements to communication between parents, health care providers, and health systems (Box 4, [3c]).

By extension, participants sometimes highlighted the need for specific improvements in health services, such as better care coordination to improve access, and even quality of life (Box 4, [4a]). One self-identified parent described an inpatient hospital experience during which her affected child was repeatedly moved from room to room, and the distress this caused (Box 4, [4b]). This experience suggests that some important, patient-centered changes to health care delivery for families living with IEMs and other rare diseases may be neither complex nor expensive.

Concerning therapeutic interventions more specifically, availability, management, and/or effectiveness were addressed by all of the participants—often offering insight into progress that has been made toward understanding and treating a particular disease. While some (though, few) participants reported a perceived lack of interventions to treat the disease of interest (Box 4, [5a]), those for whom specific treatments were available sometimes described barriers to access and/or adherence; these included costs, regulatory approvals and insurance coverage (including variability across jurisdictions), side effects, and the rigour and/or intensity of the regimen (Box 4, [5b–c]).

For those with access to treatments, challenges managing the administration of these were commonly described; nonetheless, these challenges were consistently minimized alongside gratitude for and/or the positive aspects of having treatment available (Box 3, [5d]). As it concerned the concept of treatment effectiveness, there was unsurprisingly wide variation with some treatments described as being minimally effective, while others were described as having apparent potential for transformative effectiveness (though, not necessarily in all patients) (Box 3, [5e]).

**Box 4: Struggle toward improved outcomes and interventions—example quotes**

**Table:** 

**Sub-theme 1: IEM disease communities: challenges and successes**
**[1a]** Participant (01)*: “So the challenge for these people, first of all you know, to get a diagnosis. Second of all, to get a physician who is informed about it… the kind of questions we often get… are, ‘I have this disorder’ or ‘I believe I have this disorder. I can’t get a physician to go through the process of diagnosing it. Please help me find a physician.’ They almost always end with that, ‘please find a physician’. And you know we’ve been working for over 2 years to try to get a tiny network of physicians across* [the country] *who could provide some level of support. And so at this point we have, I think, four who are aware. I think four or five physicians that would see patients with* [the disease]*.”*
**[1b]** Participant (18)*: “So yeah, I just think that right now the* [disease name] *atmosphere is very, very positive and there’s a lot of support from all the pharmaceutical companies that have treatments out there. You know, we do our best to provide what we can as well, and doctors are very giving of their time to patients so they don’t feel like they’re just pushed through the system, yeah… So, I would, I’d probably say, it’s probably more positive than negative out there! [laugh]”*
**Sub-theme 2: Role of the internet and social media**
**[2a]** Participant (02): *“… many of the adults that we know of diagnose themselves from information on the internet, and went to the doctor and said, ‘I think this is what I have.’”*
**[2b]** Participant (16): *“Yeah, and things now with social media are so different than they were… families that talk about ‘Well, when we got the diagnosis, you know, we had to go to the library. You know, we didn’t have Internet at home; we didn’t have a computer. You know, you couldn’t Google it. You had to get this big medical textbook out.’ But now families are going to Facebook and they’re finding families on Facebook before they’re finding the* [rare disease patient group]*.”*
**[2c]** Participant (13): “*If you’ve got a condition which is a rare… you may be the only patient for miles around who has that experience. But with, you know, Facebook and things like that, you can be part of a group which can have members all ‘round the world… it can help to reduce the sense of isolation that comes for families who are dealing with conditions which are poorly recognized and little understood.”*
**[2d]** Participant (09): *“… what we do find is sometimes that the newspapers don’t do us any favours. And even the Facebook groups—one person will claim that… their child’s had a treatment and it’s wonderful and they’re cured and they’re so much better and they’re going to school and they haven’t been to school for months. But we know, sort of, from the outside, that that will only be a short-term thing and that at some point they will be ill again.”*
**Sub-theme 3: Importance of communication with clinicians**
**[3a]** Participant (12): *“… of course, with the electronic age there’s a chance for parents to learn way more. You can read the journals, you can hit all the updates and abstracts, etcetera, and you may not necessarily understand it but you can write it down and then regurg*[itate]*. Yeah, so I think parents can be fantastic resources, and I think doctors… are very receptive to hearing from patients and you know at least listening to it and going, ‘Okay, that makes sense’, or ‘No, that’s not* [correct]*.’”*
**[3b]** Participant (16): *“I think just being able to admit when they don’t know enough about this disorder, you know, because not all physicians are willing to accept that usually the mum and dad know more about this particular* [disease]*… it’s very common that as a parent you are going to know more. You know, and to just be able to willingly learn, you know, from the parent and listen to them, because these kids with* [the disease] *don’t always present the same way.”*
**[3c]** Participant (03): *“… electronic records aren’t really being used as much as they should, or not at all in some places, and so you could even be at that hospital a lot and they won’t even be able to pull up all the information. So yeah, that’s an issue… people go in and they have to explain the whole situation to people who really don’t understand what they’re talking about.”*
**Sub-theme 4: Suggestions for improved health care**
**[4a]** Participant (03): *“And I mean, I think everybody who has a child with a complex medical condition like this would benefit from having more coordination. You know, being able to go into a clinic and see several specialists in one day, for example those kinds of things. And that really doesn’t happen right now. So, you know it’s a lot, and the burden of getting to all these appointments is huge.”*
**[4b]** Participant (06): *“… like in a matter of three days that* [hospital] *room had become my home, you know? And that was where I’d stay, and I never left my son’s bedside. And then when they’d tell me we were moving, like, my heart would just sink and I’d almost be in tears over it because I couldn’t handle any more change… and that was very, very difficult as a family… I know other parents have said the same thing too…”*
**Sub-theme 5: Struggles and progress in treating the disease**
**[5a]** Participant (01): *“Yes, well you know, there are no treatments. There are some things that this particular family… already found, you know, doesn’t help in their family. So there’s no treatment…”*
**[5b]** Participant (04): *“For many families, access to treatments are a struggle. Every* [jurisdiction] *is different but many patients do not have access to the formulas… foods or medication that they need to best manage* [the disease] *and minimize the neurocognitive damage.”*
**[5c]** Participant (13): *“Then there’s the demands of the treatment regime—where there is a treatment regime available. You know, a lot of inborn errors of metabolism don’t yet have a therapy, but hopefully will do as the process continues. And then there’s the uncertainties about access which can produce stress and anxiety, not just in the affected individual but also in the rest of the family. So, you know you can imagine the situation: if you know there’s a therapy coming through the licensing process, I mean, are you going to be able to access it? And are you going to be able to access it in a timely manner before your disease has progressed to the point at which, you know, you are severely constrained in what you can do?”*
**[5d]** Participant (03): *“… you know, some people travel to the hospital 3–4 h each direction. I mean that happens, right? So, some people, that’s taking 12–13 h; 14 h a week. So yeah, I mean, I would consider that onerous. But the great thing is most people don’t really see it that way. They’re grateful to have the option.”*
**[5e]** Participant (16): *“… it’s fascinating looking at those three* [children]*, you know—one who got the drug at two* [years old]*, one who got the drug at one* [year old]*, one who got the drug at, like six weeks… A huge major difference… of course, it’s so hard to demonstrate effectiveness because of the small numbers of patients, and… how come the treatment’s working on one kid and not in another? It’s just so hard, right?”*

## Discussion

A rich variety of children’s and families’ experiences, needs, and priorities were described by IEM patient group representatives with many of the concepts being represented by the key themes of coping with uncertainty, managing a child’s life transitions, and the collective struggle for improved outcomes and interventions.

Similar themes have emerged in the relatively scarce qualitative literature addressing patient and family experiences with IEMs, such as Dimond’s study of adult patients with mitochondrial disorders in which experiences that typify uncertainty were highlighted (2013). Similarly, de Ru and colleagues describe the importance of a diagnosis in easing distress associated with the ‘unknown’ in parents of children with Mucopolysaccharidosis type I (MPS I) (2012). Likewise, an accurate and timely diagnosis is documented elsewhere as an important outcome for reducing the burden of uncertainty for rare disease patients and families (Botkin et al 2010; Groft and Posada de la Paz 2010). Thus, our findings support the notion that achieving a diagnosis is itself a priority for patients and families and thus is an important component of patient-oriented care for rare diseases.

Our findings also suggest that patient self-organization is important for both mitigating uncertainty and driving progress toward improved treatments and outcomes for rare diseases. While this finding is, at least in part, a function of our participants’ roles within these groups, a growing literature examining rare disease patient self-organization reinforces it, suggesting that patients and families are less isolated and more empowered when they gather to share experiences and strategies for overcoming challenges (Aymé et al 2008; Hall 2013). Indeed, rare disease patient groups, social networks, and supportive resources have been described as critically important to the experiences of affected children and families and the progress of rare disease communities (Humphreys 2012; New York-Mid-Atlantic Consortium for Genetic and Newborn Screening Services 2009). Notably, the role of for-profit industry in these activities should not be overlooked; rare disease patient groups often form strong relationships with the well-financed pharmaceutical industry (Phillips 2013). To moderate these relationships, there may be resource-efficient ways that health care providers can support IEM patient groups with less potential for conflicted interest.

Finally, we identified challenges associated with supporting an affected child through life transitions, consistent with other studies: Ievers-Landis and colleagues report on adherence to diet for phenylketonuria (PKU) patients as being particularly problematic in adolescence (2005). Similarly, qualitative work involving patients with PKU found that the social transitions across the lifespan were particularly challenging for those affected (Vegni et al 2010), and a study of children with Niemann-Pick disease identified the ages of 10–16 as being especially challenging socially (Henderson et al 2009). In addition, there is a growing literature addressing the transition of adolescents from pediatric to adult health care (Gleeson and Turner 2012; van Staa et al 2011; Zurynski and Elliott 2013)—some of which is specific to rare-diseases, including IEM (Mutze et al 2011; Simoes et al 2014). Nonetheless, literature describing earlier life transitions, e.g., children entering the educational system (Mescon and Honig 1995), seems to have received less attention. Our findings highlight the need for additional research addressing these earlier transitions, perhaps focusing on synergies between health and educational systems in supporting children with IEM and their families.

### Strengths and limitations

One of the strengths of this study is its qualitative portrait of child and family experiences from many families living with a wide variety of IEMs. However, this breadth of experiences may come at the cost of depth, possibly overlooking unique, and very important, concerns that one or more rare disease communities may have.

The study’s breadth may have also introduced other limitations, i.e., experiences living with IEM were sought via patient organizations rather than from the families themselves. Since many participants incorporated personal insights and experiences alongside their perceptions of the experiences and priorities of others, this limitation was mitigated to some extent. Interviewing representatives of patient groups also brought a unique perspective that included, for example, insights about progress of entire rare disease communities. On the other hand, the study’s findings do not differentiate subjective, lived experiences from the objectively observed experiences of others.

Another potential limitation introduced by the study’s reliance on patient group representatives is the absence of any description of the experiences of those families for whom no patient groups exist, or those who choose not to participate in patient groups. While some reasons for group participation were described by this study’s participants (i.e., information, interconnectedness), it is not known why some families choose to participate in IEM patient groups while others choose to abstain from involvement. Studies examining reasons for patient group participation in other disease communities suggest that positive attitude and perceived control may be factors associated with patient group participation (Van Uden-Kraan et al 2011; Voerman et al 2007). Nonetheless, it must be acknowledged that any factors associated with non-participation in IEM patient groups could also be associated with unique experiences and priorities that were therefore not captured by this study.

## Conclusion

An improved understanding of the child and family experience living with IEM is an important part of ensuring that health care and other social services are prepared to adequately support affected children and families. Participant suggestions for improving care present an opportunity to consider concrete ways of providing this support, e.g., improved coordination of health care and a more collaborative, patient-centered approach to communication between clinicians and patients/families. Notably, some of these practical suggestions may also be cost-effective.

Ultimately, recognition of the challenges associated with uncertainty, managing a child’s transitions through life, and the struggle for improved interventions and outcomes can help health care providers consider other practical approaches to supporting families living with IEM, as well. For instance, clinicians can more intentionally recognize and work to reduce the uncertainty that families struggle with by being transparent in communicating what is and is not known and/or understood about the disease and its treatment. Similarly, health care providers may be able to offer support to families by suggesting coping strategies and resources for managing the child’s life transitions. Finally, the health care provider may often have a role to play in facilitating connections between families who could benefit from the support of one another.
